# Research Progress of Male Reproductive Toxicity of Chinese Materia Medicas

**DOI:** 10.1155/2019/7249679

**Published:** 2019-07-14

**Authors:** Sicong Li, Chao Li, Xiaoran Cheng, Xin Liu, Mei Han

**Affiliations:** ^1^College of Management, Beijing University of Chinese Medicine, Beijing 100029, China; ^2^Department of Emergency, China-Japan Friendship Hospital, Beijing 100029, China; ^3^College of Chinese Medicine, Beijing University of Chinese Medicine, Beijing 100029, China

## Abstract

In recent years, as the infertility rate in China has been increasing year by year and semen quality decreasing, male reproductive toxicity of drugs attracts more and more attention. There are many factors that cause male reproductive toxicity, among which Chinese materia medica is an important aspect. This article will introduce the male reproductive toxicity of Chinese materia medicas grouped by different effectivenesses such as immunosuppressant, evacuant, diuretic, cardiotonic, anti-infective drug and analgesic.

## 1. Introduction

Over the half past century, sperm counts and sperm activity parameters of semen have shown a significant downward trend. At present, Chinese economy is developing at a rapid pace. The pace of people's life has been accelerating along with living and psychological pressure increasing. Long-time use of electronic products, such as computers and mobile phones, and intake of carbonated beverages, alcohols and tobaccos all exert bad influences on quality and quantity of sperms, which leads to the increasing number of male infertility patients. Chinese materia medicas have been favored by Chinese people and become the first choice in the clinical treatment of many diseases in China. However, male reproductive toxicities of Chinese materia medica cannot be ignored [1]. When Chinese materia medica is generally used in accord with the principle of rational prescription, it is considered by the public to be of great safety. However, by means of toxicological researches, we find that some Chinese materia medicas* in vitro and in vivo* experiments exert several negative impacts on reproductive functions. After extracted and refined by modern methods, the effective and toxic components of some Chinese materia medicas are relatively enriched. While the curative effects improve, toxicities are also enhanced and new toxicities appear [2]. The targets of male reproductive toxicity in Chinese materia medica are testes, epididymides, sperms and hormone secretion systems [3]. The consequences include failures in sexual behavior, infertility, degeneration of sexual organs and decreased sperm counts and qualities [4]. Based on the classification standard of different medicinal effectivenesses, not only the pharmacological action and mechanism of each medicine, but also the manifestation and mechanism of their reproductive toxicities are introduced in this article.

## 2. Immunosuppressant

Tripterygium hypoglaucum hutch and Tripterygium wilfordii can be used as immunosuppressants [5] in the treatment of rheumatoid arthritis, Behcet's disease, membranous nephropathy and other diseases.

### 2.1. Tripterygium Hypoglaucum Hutch

The immunosuppressive mechanism of Tripterygium hypoglaucum hutch extract [6] may be related to the increase of CD+4 CD+25 T cell level and the expression of Foxp3 mRNA. The reproductive toxicity of Tripterygium hypoglaucum hutch is mainly manifested in abnormality of sperms' morphology, quantity and activity. After 30 days of taking extraction of Tripterygium hypoglaucum hutch orally [7], the pregnancy rate of mating rats decreases from 88% to 3%. The counts and activity rates of sperms decrease significantly. But testicular quality and tissue morphology change just a little. Only a slight decrease of number is found in spermatogenic epithelial cells in the seminiferous lumen. The spermatogenesis is trapped in the late stage, which prohibits spermatids from coming into being. After 60 days of taking extraction of Tripterygium hypoglaucum hutch, male rats lose fertility abilities, with motility and density of sperms in epididymides significantly decreasing and rates of abnormality significantly increasing. However, these changes can gradually recover after 20 days of withdrawal [8].

Damages to Sertoli cells' viability and DNA integrity are dependent on medicinal dosage and administration time. Germ cells and sperms are targets of Tripterygium hypoglaucum hutch's reproductive toxicity [9]. It can aggregate the RNA residues into large clumps in the seminiferous tubules. The DNA of sperm cells [10] which drop from the epididymides lumen are pushed to the nuclear membrane. Most sperms are deformed as broken head, nuclear pyknosis, and coiled tail. Fluorescence in situ hybridization (FISH) [11] shows that, after intraperitoneal injection of Tripterygium hypoglaucum hutch for a week, sperms in epididymides come into chromosomal nondisjunctions.

### 2.2. Tripterygium wilfordii

It is widely used in the treatment of rheumatoid arthritis [12], glomerulonephritis, nephrotic syndrome, lupus erythematosus and Sjogren's syndrome. It can not only inhibit T cell proliferation, spleen cell activation and antigen-induced antibody responses, but also reduce IL-2 production in spleen cell [13]. However, several components show reproductive toxicities.

#### 2.2.1. Tripterygium wilfordii Glycoside

Apoptosis of spermatogenic cells [14] can be seen in both fertile and infertile men. However, proportion of apoptotic cells differs. Excessive apoptosis may be related to diseases or drug reproductive toxicities. The percentage of apoptotic cells [15] in semen of normal fertile males is only 0.1%, while that in infertile males significantly increases, with varicocele being 1%, sperm maturation disorder being 25%, and patients who take Tripterygium wilfordii glycoside tablets 4.5 mg/kg daily being 50%. Tripterygium glycosides [16] hinder the transformation of round spermatids into long spermatids and subsequently into spermatozoa by means of destroying actin. During the spermiogenesis, actin is present in sperm cells and sperms. Actin participates in migration of central granules, occurrence of the neck, formation of fibrous sheaths and fixation of postacrosomal sheaths under nuclear membrane [17]. As a consequence, sperms' number reduces. Tripterygium wilfordii glycosides [18] can destroy a large number of sperm cells, bend the sperms' necks as well as producing teratospermia and multitailed sperms.

Tripterygium wilfordii glycosides (GRT) [19] affect reproductive-related genes and upregulate protein expression levels of mice testicular tissues' Y chromosome Ddx3y and transcriptional activators. Tripterygium wilfordii polyglycosides [20] play a toxic role in testicular Sertoli cells and spermatogenesis process by increasing FSH levels and decreasing inhibin B as well as inhibin T levels in serum.

When the reproductive system suffers from various harmful stimulants, high activity molecules, such as reactive oxygen species (ROS) and reactive nitrogen species (RNS), are produced too much in body. The degree of oxidation exceeds the clearance ability of oxidation enzyme. Imbalance exists between the oxidative system and the antioxidant system, which leads to reproductive system damage [21]. Semen antioxidant system can slow down the lipid peroxidation process of sperms and ultimately reduce the probability of sperms' premature death. The main components of antioxidant system are superoxide dismutase, vitamin E, coenzyme Q, glutathione, NADPH-dependent glutathione peroxidase and reductase equipped in sperms.

After taking Tripterygium wilfordii polyglycoside [22] at the dosage of 30mg/kg, the activity of nitrite oxide synthase (NOS) in Leydig cells decreases significantly, which results in deterioration of reproductive functions. Nitrous oxide synthase is widely distributed in male reproductive systems, which is closely related to the regulation of testicular microcirculation, testosterone secretion, spermatids' maturation and penile erection. NOS catalyzes L-arginine to produce nitrous oxide (NO) with the presence of NADPH. NO is a cell messenger molecule which exerts extensive biological functions. It plays an important role in male reproductive systems. In addition, Tripterygium wilfordii polyglycosides [23] also exert significant effects on some other enzymes: (1) the activity of alkaline phosphatase (ALP) near basement membrane of seminiferous tubule epithelium can be weakened; (2) the activity of acid phosphatase (ACP) in Sertoli cells can be enhanced; (3) the activity of 3beta-hydroxysteroid dehydrogenase (3beta-HSD) in Leydig cells of testis can be weakened; (4) the activity of lactate dehydrogenase isoenzyme-C4 (LDH-C4) is negatively correlated with the time of administration, but it can recover partially after withdrawal.

#### 2.2.2. Tripterygium wilfordii Lactone

The sperm deformity rate increases after 30 days of continuous administration of Tripterygium wilfordii lactone [24] at the dosage of 1.5g/kg, suggesting that it might have potential mutagenicity to spermatogenic cells. Tripterygium wilfordii lactone [25] has the ability to pass through blood-testis barrier and decrease the activities of SOD and GSH-Px in testicular tissues, thereby reducing the antilipid peroxidation effect, which not only destroys membranes of spermatogenic epithelial cells, supporting cells and stromal cells, but also leads to the decline of spermatogenic cells' functions and the downregulation of cell secretion growth factors. These results [26] suggest that lipid peroxidation of testicular tissues might be one of reasons why male reproductive dysfunctions appear after using Tripterygium wilfordii lactone.

#### 2.2.3. Total Alkaloids of Tripterygium wilfordii

Spontaneous apoptosis of spermatogenic cells [27] (mainly spermatogonia and spermatocytes) exists in mammals during normal spermatogenesis. Testis, as a target organ of various physical and chemical toxic medicine, can be easily affected. Apoptosis of spermatogenic cells, interstitial cells and Sertoli cells are manifestations of testicular damages. After taking total alkaloids of Tripterygium wilfordii [28] at the dosage of 10 mg/kg for six weeks, it is found that the epithelial cells of seminiferous tubules in rats' testes are damaged, the number of spermatogenic cells in seminiferous tubules decreases and the wall of seminiferous tubules becomes thinner. The damage aggravates as the dosage increases. Multinucleated giant cells come into being and are arranged in disorders [29]. When the dosage reaches to 50mg/kg, the lesion reaches the spermatocyte layer. As the number of multinucleated giant cells increases, spermatids disappear. Under electron microscopy, some sperms' mitochondrial sheaths disintegrate and the number of mitochondria decreases. The chromatin of spermatocytes is sparse. The perinuclear space becomes widened and a large number of lysosomes can be seen in the cytoplasm of spermatocytes. When the dosage reaches to 100 mg/kg, most spermatogenic cells in seminiferous tubules disappear; only sparse spermatogenic cells and supporting cells remain. When the seminiferous tubules get atrophied, necrotic and exfoliated spermatogenic cells and polynuclear giant cells can be seen. Nuclear concentration, fragmentation and dissolution necrosis changes can be observed in spermatogenic cells. Moreover, total alkaloids of Tripterygium wilfordii [30] can interfere with the synthesis of nuclear DNA in primary spermatocyte and then make mitochondria of spermatocytes swollen, cristae broken and produce visible flocculent deep-stained substances.

## 3. Evacuant

Rhubarb and cassia seed can be used in the treatment of habitual constipation and dynamic intestinal obstruction [31].

### 3.1. Rhubarb

The content of total anthraquinones in Rhubarb [32] is 1.5%~4.0%. The effective ingredients of Rhubarb in treating constipation are anthraquinone compounds. Total Anthraquinone of Rhubarb [33] can not only excite M receptors on intestinal smooth muscles, increase intestinal peristalsis, but also effectively inhibit the expression of AQP4 in rat colon and increase water content in colon. The reproductive toxicity of Rhubarb is manifested in abnormal sperm motility, quantity, and the level of reproductive hormones. Total anthraquinone [34] of Rhubarb taken at the dosage of 1g/kg for 30 days can damage the Leydig cells and Sertoli cells of Sprague Dawley (SD) rats as well as decreasing the activities of testosterone synthases and energy metabolism enzymes. As a consequence, synthesis and serum levels of testosterone reduce while serum levels of LH increase. The increase of FSH in serum is mainly due to the decrease of testosterone and the increase of compensatory regulation of hypothalamic-pituitary-testicular axis. It results in abnormal sex hormone levels in serum. Moreover, total anthraquinone of Rhubarb [35] can significantly reduce sperm counts and activity rates with testicular and epididymis index decreasing at the dosage of 1.0g/kg.

### 3.2. Cassia Seed

Anthraquinones [36] in Cassia seed can regulate the release of vasoactive intestinal polypeptide (VIP), increase the volume of intestinal contents, stimulate intestinal mucosa and increase intestinal peristalsis, thus treating constipation. After 90 days of taking Anthraquinones in Cassia seed orally [37] at the dosage of 1.0g/kg, the number of rats' spermatids reduces. Moreover, the testes get atrophied and become smaller in size. Microscopically, the seminiferous tubules get atrophied and the fibrous connective tissues get proliferated.

## 4. Diuretic

The main characteristic chemical constituent of Phytolacca is triterpenoid saponin [38], which has significant diuretic effect. However, triterpenoid saponin of Phytolacca acinosa Roxb [39] can terminate activity of all sperms in rabbit semen at the concentration of 4g/L* in vitro* test. After washing off the saponin, the sperms cannot rejuvenate. It suggests its lethal effect on rabbit sperms. At the concentration of 2.6 g/L, the total saponin of Phytolacca acinosa Roxb can terminate activity of all sperms in human semen. After washing off the saponin, human sperms cannot rejuvenate either. After reducing the concentration of saponin, spermicidal efficacy is weakened, which indicates a significant dose-effect relationship.

## 5. Cardiotonic

### 5.1. Datura Flower

Extracted from the leaves of datura flower, digitoxin [40] is mainly used for congestive heart failure. Because of its slow and lasting effect, it is suitable for long-term use in patients with chronic heart failure. However, digitoxin [41] can bring about estrogenic effects like male breast development, after long-term application at the dosage of 1mg/kg.

### 5.2. Ginseng

The ginseng saponin Rb3 [42] can enhance myocardial contractile function and can be used to treat myocardial contractile dysfunction. However, it can significantly decrease the activity of superoxide dismutase (SOD) [43], glutathione peroxidase (GSH-px), and total antioxidant capacity (T-AOC) in mice and elevate the concentration of malondialdehyde (MDA) to induce the apoptosis of spermatogenic cells.

## 6. Analgesics

### 6.1. Chinaberry Fruit

It is widely used in the treatment of abdominal pain caused by gastrointestinal spasm and hernia [44]. Obtained from petroleum ether extraction of Chinaberry fruit, Chinaberry fruit oil [45] is one of the most important effective components.* In vitro* tests, it is shown that 0.5 ml of Chinaberry fruit oil can kill sperms of SD rats within 20s. After 100 *μ*l of this oil is injected into each side of SD rats' epididymides, histological examination reveals that diameters of the testicular seminiferous tubules reduce after 6 weeks. FCM (flow cytometry) examination shows that Chinaberry fruit oil can inhibit the production of testicular spermatogenic cells, stimulate non- sperm cells and increase their anabolism.

### 6.2. Papaya

It has similar analgesic effect with Chinaberry fruit oil, but the mechanism is different. Papaya alkaloids [46] can block calcium channels in nerve tissue, inhibit calcium influx and reduce the release of pain mediators such as prostaglandins. However, the alkaloids in Carica papaya L [47] can also inhibit the activity of steroidal enzymes in rats' testes. Both oral intake and intramuscular injection of this kind of alkaloids can lead to reversible sterility in rats.

### 6.3. Radix Aconiti

The water extract of Radix Aconiti has good analgesic effect. It is mainly used to treat the pain [48] caused by rheumatoid arthritis, fracture and degenerative arthritis. However, the organ index of testes and epididymides of SD rats decreases significantly after three months administration of water extract of Radix Aconiti [49] at the dosage of 8.3 g/kg.

## 7. Hypnotic

### 7.1. Albizia Flower

Lignans from Albizia flower have sedative and hypnotic effects, that are widely used in the treatment of insomnia [50]. However, after taking lignans from Albizia flower [51] intragastrically for 60 days at the dosage of 50 mg/kg, indexes of Wistar rats' testes, epididymides, seminal vesicles and prostates with productions of spermatocytes, spermatogonia, and secondary spermatocytes decreasing significantly. Not only the area of nuclei and the number of mature supporting cells in Leydig cells, but also sperms'motility decrease significantly. The contents of protein, glycogen, cholesterol, epididymal protein and sperm vesicle fructose become less and less. The diameters of deferens get thinner significantly with the infertility rate increasing. These results suggest that the lignans extracted from flower of silktree albizzia might produce reproductive toxicity by damaging testicular cells.

## 8. Anti-Infective Drugs

### 8.1. Sophora Flavescens

Water extract of Sophora flavescens [52] has a good therapeutic effect on pneumonia caused by Staphylococcus aureus, Streptococcus hemolyticus, Escherichia coli and other pathogenic bacteria* in vivo*. The main effects are imposed on sperm density (sperm count), sperm motility, abnormal sperm rate, daily sperm production, sperm cell viability, sperm mitochondrial function and sperm acrosome integrity. Matrine in Sophora flavescens [53] could stop sperms' movement instantly at the lowest effective concentration of 0.85 g/L. Morphology of sperms shows that matrine has lethal effect on sperms. In addition, Sophora alopecuroides alkaloids [54] have spermicidal or inhibitive effects on spermatogenesis.

### 8.2. Andrographis paniculata

Known as natural antibiotic drug [55], andrographolide has good effect on bacterial upper respiratory tract infection and bacterial dysentery. This product is diterpenoid lactone compound, which is insoluble in water and can only be given orally. After 11 weeks of administration of andrographolide [56], the walls of seminiferous tubules in mice' testes become thinner, mature sperms in lumen are absent and the sperm counts significantly reduce. During the same administration period, the higher the dosage is, the more significant reduction happens. Moreover, andrographolide [57] has certain toxic effects on spermatids and testes of mice. The mechanisms may be to block the energy source of spermatids, so that spermatocytes cannot undergo meiosis process normally. Spermatogenic epitheliums in mice testes are destroyed, so that spermatogenic cells cannot be properly differentiated, resulting in abnormality of spermatids.

### 8.3. Artemisia annua

Artesunate has strong killing effect on plasmodium [58] and the ability to quickly control symptoms of dysentery. However, it is found to damage DNA double strands in Sertoli cells of mice testes. After long-term use of artesunate [59], the blood-testis barrier can be hurt as a result. Short-term use of artesunate does not have a significant effect but long-term use can reduce follicle stimulating hormone, luteinizing hormone (LH), and testosterone levels in serum.

### 8.4. Balsam Pear

A protein named MAP30 isolated from balsam pear has been shown to inhibit HIV DNA synthesis and also eliminate herpes simplex virus. However, the water extract of Momordica charantia contains an alkaline protein with a molecular weight of 34,000, which inhibits sperm activity. After Momordica charantia L. extract [60] is directly applied to the scrotum of adult mice at the dosage of 1g/kg, some of the testicular seminiferous epitheliums come into disorder. The abnormal megakaryocytes in the proximal surface of the seminiferous tubules become more and more. Large vacuoles appear in spermatids and the mitochondria get swollen. It suggests that Momordica charantia L. extract might have strong ability to stop the development of spermatids in mice, which is related to the damage of seminiferous tubules. The Momordica charantia L. extract [61] also has a detrimental effect on spermatogenic epitheliums. Spermatids are damaged at first, with early spermatocytes getting gradually involved. The acrosome membrane of spermatids becomes thicker and shorter; the number and motility of spermatids decrease. The aberration rate increases during the metamorphosis period.

## 9. Anticoagulant

Many Chinese materia medicas can be used in the treatment of miocardial infarction, pulmonary embolism, stroke and other thrombotic diseases.

### 9.1. Earthworm

Earthworm extract can reduce blood viscosity [62] and inhibit platelet aggregation. It contains fibrinolytic substances, which can promote fibrinolysis. Earthworm extract QY-III [63] (a yellow powder containing succinic acid and hyaluronic acid) has rapid killing effects on mice and human sperms* in vitro*. Its spermicidal characteristics exist in three aspects: rapid braking, agglomeration of sperms and destruction of sperm morphology. Further researches have shown that QY-III can produce many “sperm-flocs clusters”. They hinder the free movement of sperms, destroy sperms' penetrating and power device, which makes sperms lose fertilization abilities.

## 10. Antidiarrheal

Papaver shell and hedgehog skin are often used to treat diarrhea caused by irritable bowel syndrome, hepatitis, inflammatory bowel diseases, chronic pancreatitis and tumors in digestive tract.

### 10.1. Pericarpium Papaveris

It can cause difficulty in ejaculation [64] and erection. Long-term use of morphine reduces male testosterone secretion and deteriorates secondary sexual characteristics.

### 10.2. Hedgehog

Saponins isolated from hedgehog skin can stabilize the acrosome membrane of sperms [65], which hinder the release of acid hydrolase and protease in sperms. Failure or obstruction of acrosome enzyme release can hinder the fertilization of eggs, thereby playing an antifertility role.

## 11. Antigout Drugs

### 11.1. Meadow Saffron

Colchicine, an alkaloid originally extracted from the liliaceous plant meadow saffron, can be used in the treatment of acute attacks of gouty arthritis to prevent joint damage caused by urate. Colchicine [66] cannot only inhibit sperms' motility, induce sperms' apoptosis, but also reduce the secretion of serum testosterone, follicle stimulating hormone (FSH) and luteinizing hormone (LH). Infertility happens when reproductive-related hormones are at abnormal level.

## 12. Antineoplastic Drugs

### 12.1. Solanine

It can significantly prolong the survival time of H22 tumor-bearing mice [67]* in vivo*.* In vitro*, solanine exerts strong inhibitory effects on the proliferation of HepG2 cells, PC-3 cells of prostate cancer, PANC-1 cells of pancreatic cancer, A2058 cells of human melanoma, HeLa cells of cervical cancer and U251 cells of human glioma. However, solanine [68] can decrease the activity of superoxide dismutase (SOD), succinate dehydrogenase (SDH) in testicular tissues and the content of glutathione (GSH) in testicular tissues. It results in the increase of free radicals in testes, abnormality in respiratory chain, blockage of tricarboxylic acid cycle, increase in osmotic pressure of the mitochondrial matrix and oxidative damage of the mitochondria [69].

### 12.2. Gossypol

Often used in the treatment of lymphoma and leukemia, gossypol [70] can induce cancerous cells apoptosis. After taking orally 30~60mg/kg gossypol [71] for five weeks, the testicular seminiferous tubules of rats relatively reduce, the number of spermatogenic cells significantly reduce and a small number of multinucleated giant cells can be seen. There are rare mature sperms in the seminiferous lumen. In some rats, spermatogenic epithelial loss and shedding of immature spermatogenic cells can be observed. In epididymides, gossypol can make the acrosome of sperms go into rupture and the head of sperms to break apart from their tails. The mitochondrial spiral sheaths in the middle of the sperms come into disorder and become swollen. Moreover, gossypol [72] can significantly inhibit acrosomal enzymes' activity in sperms, thus reducing fertilization opportunities. These pathological changes can recover automatically after drug withdrawal for two months.

### 12.3. Glycyrrhiza Uralensis Fisch

It can inhibit the proliferation of cancer cells and induce apoptosis of cancer cells [73] by inhibiting nucleotide reductase and reducing the activity of DNA synthesis rate-limiting enzyme. After 60 days of taking 450mg/kg Glycyrrhiza uralensis Fisch extract [74], the testicular tissue structures of rats undergo remarkable deterioration. Atrophy of seminiferous tubules and hyperplasia of connective tissues can be seen. The number of intermediate cells in connective tissues significantly reduces. The Glycyrrhiza uralensis Fisch [75] can lead to testicular and penile atrophy, sexual apathy and reduction in times of chasing female rats and sexual intercourse.

## 13. How to Look upon the Reproductive Toxicity of Chinese Materia Medicas Reasonably

Most objects of the researches mentioned above are purified single components. Traditional Chinese medical therapy does not use these single components for clinical treatment but use several medicinal materials that are combined together. One traditional Chinese medicine decoction can contain thousands of different monomers. Therefore, it is improper to abandon the use of Chinese materia medicas simply because of reproductive toxicities of some certain monomer components. In practical clinical applications, Chinese medicine treatment is often used in combination with two or more medicinal materials. In this process, the monomer components with reproductive toxicity often undergo chemical reactions, which lead to quantitative or qualitative changes. As long as Chinese materia medicas are used in scientific and reasonable ways [76], the pharmacological effects can be enhanced, the toxicity and adverse reactions can be reduced. Moreover, Chinese medicine treatment is guided by the basic theory of traditional Chinese medicine, which emphasizes on dialectical analysis of the whole condition. As long as the medical treatment is under the guidance of basic theories of traditional Chinese medicine, the reproductive toxicities can be under control. Discussions on the reproductive toxicities must pay attention to the course of treatment and dosage. The clinical application of conventional Chinese medicine is usually no more than 7 days, while the administration time of drugs in the basic researches above has far exceeded 7 days. Moreover, the dosage is neither a conventional therapeutic dosage nor a maximum safe dosage.

## 14. Summary

This article describes not only direct pathways in which Chinese materia medicas cause infertility by killing sperms and damaging testicular tissues, but also indirect ways of affecting reproductive-related hormones. However, we cannot abandon the use of Chinese materia medicas because of some toxicity reports. The reproductive toxicities can also be used for clinical treatment. As long as doctors use them reasonably, toxic ingredients can be also therapeutic ingredients. For example, trichosanthin [77] has the effect of terminating mid-late pregnancy; therefore trichosanthin has been used for maternal induction of labor and shortening the labor process. It is the same for male reproductive toxicity. Chemical components [78] that kill sperms can be used for contraception at therapeutic dosage. Therefore, we must take a dialectical view of the reproductive toxicities of Chinese materia medicas, do our best to avoid adverse reactions and make unremitting efforts to improve safety of clinical applications.

## Data Availability

The data used to support the findings in this study are included within the article.
